# Diagnostic value of cerebrospinal fluid Aβ ratios in preclinical Alzheimer’s disease

**DOI:** 10.1186/s13195-015-0159-5

**Published:** 2015-12-18

**Authors:** Katarzyna Adamczuk, Jolien Schaeverbeke, Hugo M. J. Vanderstichele, Johan Lilja, Natalie Nelissen, Koen Van Laere, Patrick Dupont, Kelly Hilven, Koen Poesen, Rik Vandenberghe

**Affiliations:** Laboratory for Cognitive Neurology, KU Leuven, Herestraat 49, 3000 Leuven, Belgium; Alzheimer Research Centre KU Leuven, Leuven Institute of Neuroscience and Disease, Herestraat 49, 3000 Leuven, Belgium; ADx NeuroSciences, Technologiepark 4, 9052 Gent, Belgium; GE Healthcare, Björkgatan 30, 751 25 Uppsala, Sweden; Nuclear Medicine and PET, Department of Surgical Sciences, Uppsala University, 751 85 Uppsala, Sweden; Department of Psychiatry, Oxford University, Oxford, OX3 7JX UK; Nuclear Medicine and Molecular Imaging Department, KU Leuven and University Hospitals Leuven, Herestraat 49, 3000 Leuven, Belgium; Laboratory for Neuroimmunology, KU Leuven, Herestraat 49, 3000 Leuven, Belgium; Laboratory for Molecular Neurobiomarker Research, KU Leuven, Herestraat 49, 3000 Leuven, Belgium; Laboratory Medicine, UZ Leuven, Herestraat 49, 3000 Leuven, Belgium; Neurology Department, University Hospitals Leuven, Herestraat 49, 3000 Leuven, Belgium

## Abstract

**Introduction:**

In this study of preclinical Alzheimer’s disease (AD) we assessed the added diagnostic value of using cerebrospinal fluid (CSF) Aβ ratios rather than Aβ42 in isolation for detecting individuals who are positive on amyloid positron emission tomography (PET).

**Methods:**

Thirty-eight community-recruited cognitively intact older adults (mean age 73, range 65–80 years) underwent ^18^F-flutemetamol PET and CSF measurement of Aβ1-42, Aβ1-40, Aβ1-38, and total tau (ttau). ^18^F-flutemetamol retention was quantified using standardized uptake value ratios in a composite cortical region (SUVR_comp_) with reference to cerebellar grey matter. Based on a prior autopsy validation study, the SUVR_comp_ cut-off was 1.57. Sensitivities, specificities and cut-offs were defined based on receiver operating characteristic analysis with CSF analytes as variables of interest and ^18^F-flutemetamol positivity as the classifier. We also determined sensitivities and CSF cut-off values at fixed specificities of 90 % and 95 %.

**Results:**

Seven out of 38 subjects (18 %) were positive on amyloid PET. Aβ42/ttau, Aβ42/Aβ40, Aβ42/Aβ38, and Aβ42 had the highest accuracy to identify amyloid-positive subjects (area under the curve (AUC) *≥* 0.908). Aβ40 and Aβ38 had significantly lower discriminative power (AUC = 0.571). When specificity was fixed at 90 % and 95 %, Aβ42/ttau had the highest sensitivity among the different CSF markers (85.71 % and 71.43 %, respectively). Sensitivity of Aβ42 alone was significantly lower under these conditions (57.14 % and 42.86 %, respectively).

**Conclusion:**

For the CSF-based definition of preclinical AD, if a high specificity is required, our data support the use of Aβ42/ttau rather than using Aβ42 in isolation.

## Introduction

Preclinical [1, 2], or asymptomatic [3], Alzheimer’s disease (AD) is characterized by the presence of AD-related pathophysiological processes in the absence of cognitive deficits. Evidence of brain amyloidosis is a requirement common to all three National Institute on Ageing–Alzheimer’s Association (NIA–AA) stages of preclinical AD [1] and is also a defining feature of the asymptomatic at risk for AD state according to the International Working Group IWG-2 criteria [3]. This can be detected directly in vivo by means of either amyloid-beta (Aβ) protein quantification in cerebrospinal fluid (CSF) or positron emission tomography (PET) amyloid imaging [1, 3–5].

Apart from Aβ1–42, other Aβ isoforms (e.g., Aβ1–40, Aβ1–38) have evoked interest from a clinical-diagnostic perspective, as either a separate biomarker tool or when combined (ratio) with Aβ1–42 [6–8]. Using ratios of Aβ isoforms (Aβ1–42/Aβ1–38, Aβ1–42/Aβ1–40) may have added value for the discrimination between AD and normal pressure hydrocephalus [9], cerebral amyloid angiopathy [10], frontotemporal dementia [11], and Lewy body dementia [12], and also between mild cognitive impairment (MCI) due to AD versus non-AD MCI [13]. In cognitively intact individuals, Aβ38 or Aβ40 does not correlate with amyloid PET positivity, in contrast with Aβ42 [5, 14].

In this study of preclinical AD, we assessed the added value of using ratios of Aβ42 to other C-terminal Aβ isoforms or to total tau (ttau) for discriminating amyloid-positive versus amyloid-negative cognitively intact healthy controls, with an autopsy-validated ^18^F-flutemetamol cutoff score [15] as standard of truth. The cutoff value was derived from the ^18^F-flutemetamol phase 3 study using a binarized measure of postmortem brain neuritic plaque density [16] (overall mean Bielschowsky score below or above 1.5 [15]). We also explored the diagnostic value of the Aβ38 and Aβ40 isoforms on their own.

For design of clinical trials in preclinical AD, the data presented may inform the decision on which CSF parameter to select for study eligibility based on its equivalence to an amyloid-PET-based definition. We not only provide the parameters providing optimal balance between sensitivity and specificity but also the parameters that provide an acceptable sensitivity for a fixed high specificity. Specificity may receive more weight in trials in preclinical AD because the definition of the target population often heavily relies on the biomarker value, healthy volunteers are exposed to potential adverse effects of study drugs for a long duration, and positive evidence for the presence of the study target increases the likelihood of success. Sensitivity will mainly determine the number needed to screen, and will therefore impact on the cost.

## Methods

### Participants

Thirty-eight cognitively intact older controls (mean age 73 years, standard deviation (SD) 5 years; Table 1) were recruited prospectively and consecutively, from 10 September 2012 until 4 April 2014, through advertisement in local newspapers and through websites for seniors, asking for healthy volunteers between 65 and 80 years of age for participation in a scientific study at the University Hospital Leuven, Belgium, involving brain imaging (sic). At screening, subjects underwent a detailed interview about medical history, a Mini Mental State Examination (MMSE), a Clinical Dementia Rating (CDR), blood sampling, and a conventional neuropsychological assessment. Inclusion criteria were age 65–80 years, MMSE *≥ *27, CDR = 0, and normal test scores on neuropsychological assessment according to the published norms adapted for age, gender, and education. Among the exclusion criteria were a neurological or psychiatric history and focal brain lesions on structural magnetic resonance imaging (MRI). Subjects who fulfilled all criteria underwent both ^18^F-flutemetamol PET and lumbar puncture. The target sample size of the PET-plus-CSF cohort was 40 but two subjects dropped out after the PET scan and prior to the lumbar puncture, giving a final sample size of 38.

**Table 1:** Demographics and CSF biomarker concentrations

Gender (male/female)	22/16	LVF (number of words)	36.0 (10.8, 17–64)
Age (years)	73 (4.7, 65–80)	RPM (/60)	36.1 (9.8, 15–53)
Education (years)	13.4 (3.1, 8–20)	TMT B/A	2.4 (0.5, 1.5–3.8)
APOE ε4 carriers (*n*)	19 (50 %)	Aβ38 (pg/ml)	2401 (654, 1057–3505)
BDNF *met* carriers (*n*)	20 (53 %)	Aβ40 (pg/ml)	8933 (2456, 3640–13273)
MMSE (/30)	28.9 (1.0, 27–30)	Aβ42 (pg/ml)	996 (430, 351–1859)
AVLT TL (/75)	46.2 (8.4, 31–69)	ttau (pg/ml)	360 (134, 126–660)
AVLT DR (/15)	9.8 (2.5, 5–14)	Aβ42/Aβ38	0.412 (0.119, 0.136–0.596)
AVLT %DR	83.7 (11.7, 55–108)	Aβ42/Aβ40	0.110 (0.030, 0.044–0.148)
BNT (/60)	54.2 (4.2, 41–60)	Aβ42/ttau	3.015 (1.246, 0.749–5.128)
AVF (number of words)	24.0 (5.5, 14–40)	Amyloid+ (*n*)	7 (18 %)

Data presented as mean (standard deviation, range)

*Aβ* amyloid beta, *APOE* apolipoprotein E, *AVF* Animal Verbal Fluency Test, *AVLT* Rey Auditory Verbal Learning Test, *BDNF* brain-derived neurotrophic factor, *BNT* Boston Naming Test, *CSF* cerebrospinal fluid, *DR* delayed recall, *LVF* Letter Verbal Fluency Test, *MMSE* Mini Mental State Examination, *RPM* Raven’s Progressive Matrices, *TL* total learning, *TMT* Trail Making Test (part B divided by part A), *ttau* total tau

This PET-plus-CSF cohort belonged to a larger cohort of healthy older controls undergoing ^18^F-flutemetamol PET (target sample *n* = 180, recruited until time of writing *n* = 172) [17, 18]. The other subjects in this larger cohort did not undergo lumbar puncture per protocol. The primary aim of the full cohort was to investigate the interaction between brain-derived neurotrophic factor (BDNF) and apolipoprotein E (APOE) genetic polymorphisms on amyloid deposition and functional reorganization [17, 18]. The inclusion and exclusion criteria for the full cohort were identical to those of the PET-plus-CSF cohort apart from the age range (50–80 years for the full cohort). At inclusion, participants of the full cohort were stratified per age bin for two genetic factors: BDNF (*met* allele at codon 66 present or absent) and APOE (ε4 allele present or absent). The cells of this 2 × 2 factorial design were prospectively matched for number of cases, APOE and BDNF genetic status, age, sex, and education.

The PET-plus-CSF cohort (*n* = 38) did not differ from the remaining subjects (*n* = 134) with regards to sex, education, number of APOE ε4 carriers or BDNF *met* carriers, the presence of subjective memory complaints (29 % in each of the two groups), or neuropsychological test scores (*P* > 0.23). The CSF cohort was significantly older than the remaining subjects (mean age 73 years vs. mean age 67 years, *P* < 0.0001). The proportion of amyloid-positive cases did not differ significantly between the CSF-plus-PET cohort (18 %) and the remaining subjects (12 %) (*P* = 0.23).

The protocol (EudraCT: 2009-014475-45) was approved by the Ethics Committee University Hospitals Leuven, Belgium. Written informed consent was obtained from all subjects in accordance with the Declaration of Helsinki.

### Amyloid PET

^18^F-flutemetamol PET was acquired on a 16-slice Siemens Biograph PET/CT scanner (Siemens, Erlangen, Germany). The tracer was injected as a bolus into an antecubital vein (mean activity 150 MBq, SD 5 MBq, range 134–162 MBq). Scan acquisition started 90 minutes after tracer injection and lasted for 30 minutes [17–20]. Prior to PET acquisition, a low-dose computed tomography scan of the head was performed for attenuation correction. Random and scatter correction were applied. The PET summed image was spatially normalized to Montreal Neurological Institute (MNI) space using a fully automated PET-only method [21]. On the basis of spatially normalized images (voxel size 2 × 2 × 2 mm^3^), standardized uptake value ratios (SUVR) were calculated with cerebellar gray matter as the reference region. The mean SUVR value was calculated in a composite cortical region (SUVR_comp_) [15]. The composite cortical region and the cerebellar gray matter reference region were defined as a combination of narrow automated anatomic labeling-type regions [22] outlined on the ICBM-152 template masked with a gray matter probability mask [15]. Images were analyzed by an experienced medical imaging specialist blinded to all study information.

To estimate the SUVR_comp_ cutoff value for detecting amyloid positivity in vivo using the described method, receiver operating curve (ROC) analysis was performed by Thurfjell et al. [15] on an independent dataset of 68 SUVR_comp_ values (quantified based on the already described method) with the autopsy results as a standard of truth. The autopsy data were classified following Vemuri’s modification of the Consortium to Establish a Registry for AD criteria [16, 23]. Eight cortical regions (precuneus, midfrontal cortex, superior temporal cortex, middle temporal cortex, inferior parietal cortex, anterior cingulate gyrus, posterior cingulate gyrus, and primary visual cortex) were scored using an overall mean Bielschowsky score: 0 = no plaques, 1 = one to five plaques, 2 = six to 19 plaques, 3 = 20 or more plaques. If the mean Bielschowsky score was *> *1.5 in at least one region, the brain was classified as amyloid-positive; if all regions scored *≤ *1.5, the brain was classified as amyloid-negative. The resulting SUVR_comp_ cutoff value was 1.57 [15].

### Lumbar puncture and CSF analysis

Lumbar punctures were carried out at the L4/5 level in the morning (10 a.m.–2 p.m.) and collected in polypropylene tubes (total volume 15 ml, Greiner Bio-one Cellstar; VWR, Leuven, Belgium), discarding 1 ml to avoid traumatic blood contamination. Samples were centrifuged within 30 minutes after collection (2600 rpm, 10 minutes, 4 °C). After centrifugation, supernatants were transferred into polypropylene tubes and from there aliquoted in 1.5 ml polypropylene tubes (1 ml volume CSF/tube; Kartell, Noviglio, Italy). Samples were stored at –80 °C until batch analysis. Our primary analysis was based on the EUROIMMUN single analyte enzyme-linked immunosorbent assays (ELISA) (EUROIMMUN, Lübeck, Germany) of CSF Aβ1–42, Aβ1–40, Aβ1–38, and ttau. The assays were performed at ADx Ghent, Belgium by two experienced laboratory technicians blinded to all study information. The Aβ assays quantify the full length of the C-terminus-specific Aβ isoforms (Aβ1-specific assay format). The tau assay is designed with a capture antibody towards the central region and one monoclonal antibody with an epitope at the amino-terminus of the protein. The assay design includes lyophylized recombinant proteins as calibrators, run-validation control samples (calibrators added to a phosphate-buffered solution), as well as a qualification panel to evaluate the analytical performance(s) in the laboratory. These novel immunoassays are free from matrix interference and their intra-assay reproducibility has a coefficient of variation ≤ 5.0 % with an inter-assay reproducibility ≤ 8.3 % [24].

As a secondary analysis, we verified our results using the INNOTEST ELISA for Aβ1–42, ttau, and ^181^phospho-tau (ptau) (Fujirebio Europe, Ghent, Belgium). The assays were performed at the Laboratory Medicine Department of UZ Leuven, Belgium, in a ISO-15189 and Joint Commission International accredited environment by an expert technician blinded to all study information. The assay design included ready-to-use recombinant proteins as calibrators, run-validation control samples, and internal quality controls samples (for which target value and acceptance criteria were established in the routine setting of AD biomarker quantification).

### Statistical analysis

In the primary analysis, which was based on the EUROIMMUN assays, we compared the diagnostic accuracy of different CSF Aβ isoforms, their ratios, ttau, and Aβ42/ttau to detect amyloid-positive older individuals. We used a ROC analysis with CSF analytes as variables of interest and ^18^F-flutemetamol positivity defined based on the autopsy-derived SUVR_comp_ cutoff value as a classifier. We also evaluated whether case classification changed when we varied the cutoff value by ±1.5 %, corresponding to the test–retest variability estimated for SUVR_comp_ [20]. The highest Youden index (sensitivity + specificity – 1) was used to estimate the optimal ROC cutoff values. Statistical differences between ROCs were evaluated according to the method of DeLong et al. [25] for pairwise ROC comparisons. Correction for multiple comparisons (*n* = 21) was performed with the Bonferroni method. The Bonferroni corrected threshold for significance was *P* < 0.002, corresponding to *P*_corrected_ < 0.05.

Depending on the study, a high specificity may be desirable even if this implies a loss of sensitivity. We therefore also evaluated sensitivities and cutoff values at a fixed prespecified specificity of 90 % and 95 %, respectively. We evaluated whether this changed case classification significantly (McNemar test).

As a secondary analysis, we performed ROC analyses based on the INNOTEST assay of Aβ_42_, ttau, and ptau and statistically compared the areas under the curves (AUCs) between the two types of assays. We also compared the AUCs between the different INNOTEST measures and determined the sensitivity and percentage of correct classifications at a fixed specificity of 90 % and 95 %.

As a further secondary analysis, we evaluated the continuous relationship between the different CSF analytes and ^18^F-flutemetamol SUVR_comp_ values. We tested whether a linear, polynomial (quadratic), exponential, or hyperbolic relation fitted best to these data. The model assumptions were assessed by evaluating normality and homoscedasticity of residuals with q–q plots and plots of residuals versus fitted values. The best fitting model was selected based on the Akaike information criterion (AIC), which is a measure of model fit. A lower AIC indicates a better fit. CSF analytes were used as dependent variables and ^18^F-flutemetamol SUVR_comp_ as an independent variable.

Statistical analyses were performed in R version 3.1.1 (https://www.r-project.org) and MedCalc version 14.8.1 (https://www.medcalc.org).

## Results

**Fig. 1:**
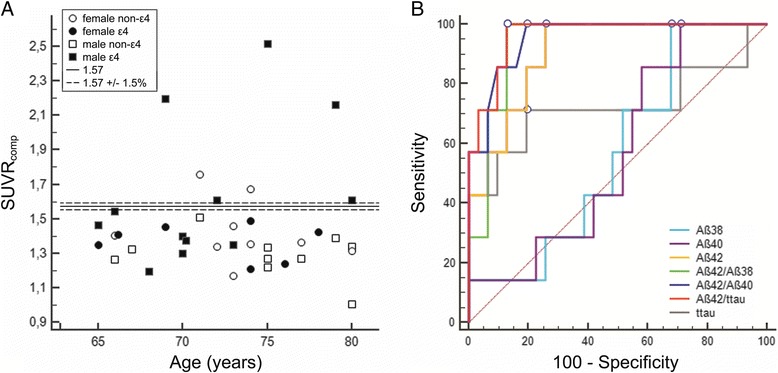
Distribution of ^18^F-flutemetamol SUVR_comp_ values and ROCs for different CSF analytes. **a** Distribution of ^18^F-flutemetamol SUVR_comp_ values according to age, sex, and APOE genotype. *Solid line* 1.57 SUVR_comp_ cutoff value; *dashed line* 1.57 SUVR_comp_ cutoff value ±1.5 % corresponding to a test–retest variability for SUVR_comp_ [20] (1.594 and 1.547). **b** ROCs for different CSF analytes, with ^18^F-flutemetamol positivity as classifier. *Dots* optimal cutoff values for each analyte, corresponding to the highest Youden index. *Aβ* Amyloid beta, *SUVR*_*comp*_ standardized uptake value ratios in composite cortical region, *ttau* total tau

Based on the autopsy-confirmed ^18^F-flutemetamol SUVR_comp_ cutoff value, seven out of 38 subjects (18 %) were assigned to the amyloid-positive category (Fig. 1a). Case assignment did not change when we varied the cutoff value according to the known test–retest replicability.

APOE ε4 carriers had significantly lower values of Aβ42, Aβ42/ttau, Aβ42/Aβ40, and Aβ42/Aβ38 than ε4 noncarriers (*P* < 0.003). CSF analyte concentrations did not differ between BDNF *met* carriers and noncarriers (*P* > 0.23).

Aβ42/ttau, Aβ42/Aβ40, Aβ42/Aβ38, and Aβ42 discriminated between ^18^F-flutemetamol-positive and ^18^F-flutemetamol-negative subjects with high accuracy (AUC ≥ 0.908; Table 2, Fig. 1b). Aβ38, Aβ40, and ttau showed a lower discriminative power with AUC ≤ 0.724 (Table 2). Aβ42/ttau, Aβ42/Aβ40, and Aβ42 had significantly higher AUCs than Aβ38 or Aβ40 alone (Table 2, *P < *0.003). Aβ42/Aβ38 had significantly higher AUCs than Aβ40 (*P =* 0.002). There was no significant difference between the ratios Aβ42/ttau, Aβ42/Aβ40, and Aβ42/Aβ38, on the one hand, and Aβ42 alone, on the other (Table 2, *P > *0.32). The AUCs of the three ratios were not statistically different from each other (Table 2, *P > *0.30).

**Table 2:** Diagnostic performance of different CSF analytes with ^18^F-flutemetamol PET as autopsy-validated standard of truth (EUROIMMUN assay)

	AUC	SE	95 % CI	Cutoff^a^	Sensitivity (%)	Specificity (%)	Correctly classified^b^ (%)
Aβ38	0.571	0.111	0.401–0.730	2909	100	32.26	45
Aβ40	0.571	0.112	0.401–0.730	10738	100	29.03	42
Aβ42*†	0.908	0.051	0.769–0.977	745	100	74.19	79
ttau	0.724	0.148	0.555–0.856	436	71.43	80.65	76
Aβ42/Aβ38*	0.935	0.039	0.806–0.989	0.332	100	87.10	89
Aβ42/Aβ40*†	0.954	0.033	0.832–0.995	0.096	100	80.65	84
Aβ42/ttau*†	0.963	0.028	0.846–0.998	2.006	100	87.10	89

Analyte concentrations are described as pg/ml or calculated as ratios between concentrations of two analytes

Statistically significant differences of AUCs between analytes: *P_corrected_ < 0.05 compared with Aβ40; †*P*_corrected_ < 0.05 compared with Aβ38. No other differences of AUCs were found

^a^Cutoff value corresponding to the highest Youden index

^b^Percentage of positively classified cases based on the CSF cutoff compared with amyloid PET classification

*Aβ* amyloid beta, *AUC* area under the receiver operating characteristic curve, *CI* confidence interval, *CSF* cerebrospinal fluid, *PET* positron emission tomography, *SE* standard error, *ttau* total tau

When specificity was fixed at 90 %, Aβ42/ttau and Aβ42/Aβ40 had the highest sensitivity and Aβ42/Aβ38 the second highest sensitivity (Table 3). All three Aβ isoforms (Aβ42, Aβ40, Aβ38) used on their own detected significantly fewer amyloid PET-positive cases when specificity was fixed a priori at 90 % than when the cutoff value was based on the highest Youden index (Table 3), indicative of a significant loss in sensitivity. This was not the case for Aβ42/ttau, Aβ42/Aβ40, and Aβ42/Aβ38 ratios and ttau (Table 3).

**Table 3:** Clinical accuracy: estimated sensitivities and cutoff values at a fixed specificity of 90 % or 95 % (EUROIMMUN assay)

	Sensitivity (%)	95 % CI	Cutoff value	Difference^a^ (%)	*P* value^b^	Correctly classified^c^ (%)
Specificity of 90 %						
Aβ38	14.29	0.00–71.43	1446	65.79	<0.0001	79
Aβ40	14.29	0.00–71.43	5602	65.79	<0.0001	76
Aβ42	57.14	0.00–100.00	546	21.05	0.008	84
ttau	57.14	14.29–100.00	471	10.53	0.125	82
Aβ42/Aβ38	71.43	0.00–100.00	0.268	7.89	0.250	87
Aβ42/Aβ40	85.71	14.29–100.00	0.074	10.53	0.125	89
Aβ42/ttau	85.71	14.29–100.00	1.852	5.26	0.500	89
Specificity of 95 %						
Aβ38	14.29	0.00–71.43	1342	68.42	<0.0001	82
Aβ40	14.29	0.00–71.43	5254	71.05	<0.0001	82
Aβ42	42.86	0.00–85.71	493	28.95	0.001	87
ttau	42.86	0.00–85.71	539	18.42	0.016	84
Aβ42/Aβ38	28.57	0.00–71.43	0.251	21.05	0.008	84
Aβ42/Aβ40	57.14	8.62–85.71	0.067	21.05	0.008	89
Aβ42/ttau	71.43	28.57–100.00	1.415	13.16	0.063	92

Analyte concentrations are described as pg/ml or calculated as ratios between concentrations of two analytes

^a^Percentage of subjects who were classified differently based on the cutoff values from fixed specificities compared with the cutoff values corresponding to the highest Youden index (Table 2)

^b^Significance for the “Difference”^c^Percentage of positively classified cases based on the CSF cutoffs from fixed specificities compared with amyloid PET classification

*Aβ* amyloid beta, *CI* confidence interval, *ttau* total tau

When specificity was fixed at 95 %, Aβ42/ttau had the highest sensitivity (Table 3). All Aβ isoforms, ttau, and all ratios detected significantly less amyloid-positive cases when the specificity was fixed a priori at 95 % compared with the highest Youden index-based cutoff value, with one exception—namely the ratio Aβ42/ttau (Table 3). At a specificity of 95 %, the number of amyloid PET-positive cases detected based on the ratio Aβ42/ttau did not differ significantly from the number detected based on the highest Youden index-based cutoff value, although it was numerically lower.

As a secondary analysis, we compared the AUCs between two types of assays, EUROIMMUN and INNOTEST. The AUCs for Aβ42, ttau, and Aβ42/ttau did not differ between the EUROIMMUN and INNOTEST assays (Aβ42, *P* = 0.33; ttau, *P* = 0.91; and Aβ42/ttau, *P* = 0.25) (Tables 2 vs. 4). When we compared the AUCs between the different INNOTEST measures, the AUC for Aβ42/ttau differed significantly from the AUC for ttau (uncorrected *P* = 0.0172) or ptau (uncorrected *P* = 0.0096). When specificity was fixed at 90 %, Aβ42 and Aβ42/ttau had the highest sensitivity (Table 4). When specificity was fixed at 95 %, Aβ42/ttau had the highest sensitivity (Table 4).

**Table 4:** Diagnostic performance of different CSF analytes measured with the INNOTEST assay for Aβ42, ttau, and ptau at an optimal specificity and at a specificity fixed at 90 % or 95 %

	AUC	SE	95 % CI	Cutoff^a^	Sensitivity (%)	Specificity (%)	Correctly classified^b^ (%)
Aβ42	0.935	0.0394	0.806–0.989	853	100	83.87	87
ttau	0.733	0.132	0.565–0.863	352	71.43	77.42	76
ptau	0.675	0.139	0.504–0.818	86	42.86	93.55	84
Aβ42/ttau	0.880	0.0878	0.734–0.963	2.258	85.71	90.32	89
Specificity of 90 %	Sensitivity (%)		95 % CI	Cutoff^a^	Difference^c^ (%)	*P* value^d^	Correctly classified^b^ (%)
Aβ42	85.71		11.54–100.00	798	7.90	0.25	89
ttau	57.14		14.29–100.00	465	10.53	0.125	82
ptau	42.96		0.00–85.71	87	5.26	0.5	79
Aβ42/ttau	85.71		28.57–100.00	2.263	0	1	89
Specificity of 95 %	Sensitivity (%)		95 % CI	Cutoff^a^	Difference^c^ (%)	*P* value^d^	Correctly classified^b^ (%)
Aβ42	42.86		4.05–100.00	672	21.05	0.008	87
ttau	14.29		0.00–85.71	566	23.69	0.004	82
ptau	28.57		0.00–71.43	94	2.63	1	82
Aβ42/ttau	71.43		8.71–100.00	2.093	7.90	0.25	92

Analyte concentrations are described as pg/ml or calculated as ratios between concentrations of two analytes

^a^Cutoff value corresponding to the highest Youden index

^b^Percentage of positively classified cases based on the CSF cutoff value compared with amyloid PET classification

^c^Percentage of subjects who were classified differently based on the cutoff values from fixed specificities compared with the cutoff values corresponding to the highest Youden index

^d^Significance for the “Difference”

*Aβ* amyloid beta, *AUC* area under the receiver operating characteristic curve, *CI* confidence interval, *CSF* cerebrospinal fluid, *PET* positron emission tomography, *ptau* ^181^phospho-tau, *SE* standard error, *ttau* total tau

**Fig. 2:**
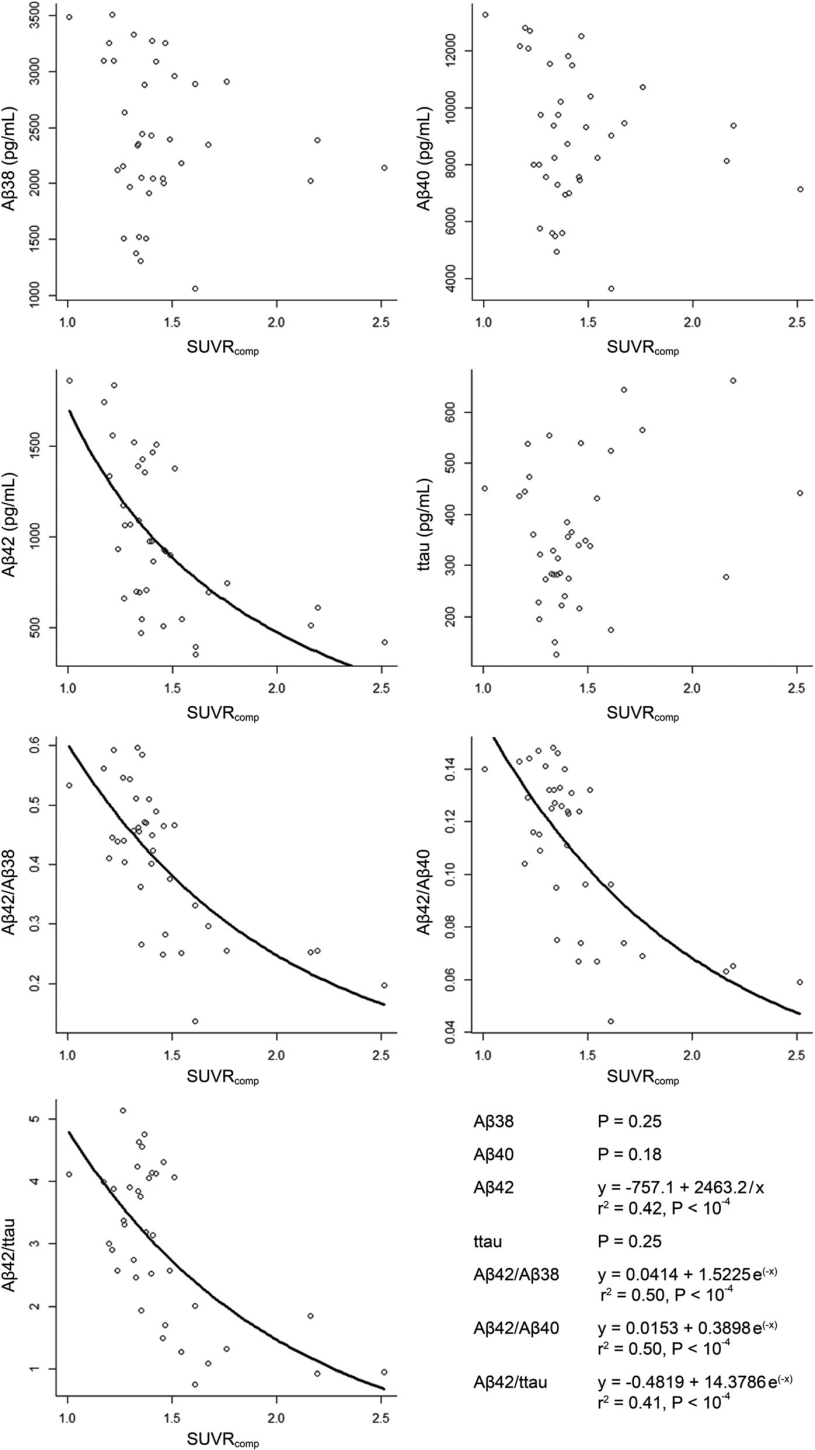
Associations between the different CSF analytes and ^18^F-flutemetamol SUVR_comp_. *Black lines* fitting of the model, shown only for the significant correlations. *Aβ* Amyloid beta, *SUVR*_*comp*_ standardized uptake value ratios in composite cortical region, *ttau* total tau

Four CSF analytes—Aβ42/ttau, Aβ42/Aβ40, Aβ42/Aβ38, and Aβ42—showed a significant correlation with the ^18^F-flutemetamol SUVR_comp_ values (Fig. 2). The linear model was rejected because it did not satisfy assumptions of the model. The hyperbolic model fitted best to the relationship between Aβ42 and ^18^F-flutemetamol SUVR_comp_. The relationships between ^18^F-flutemetamol SUVR_comp_ and Aβ42/ttau, Aβ42/Aβ40, and Aβ42/Aβ38 were best described by the exponential model. However, differences between the models were small. There was no correlation between ^18^F-flutemetamol SUVR_comp_ values and Aβ38, Aβ40, and ttau (Fig. 2).

## Discussion

Overall, when sensitivity and specificity were combined, the ability to discriminate amyloid-positive from amyloid-negative cognitively healthy older adults was comparable between Aβ42 on its own and the ratio of Aβ42 over the isoforms examined or over ttau. However, when a high specificity of 90–95 % was imposed as a criterion, the sensitivity of Aβ42 alone diminished to 43–57 %. The sensitivity of the ratio over Aβ40 was acceptable at a specificity of 90 % (86 %), but at a specificity of 95 % the sensitivity decreased to 57 %. Under these requirements, the ratio over ttau was the only measure which retained an acceptable sensitivity (71–86 %). A high specificity would for instance be desirable if the potential benefit of a study drug depends on the amyloid positivity of cognitively normal subjects and the study drug has potentially noxious effects or a high cost. A favorable tradeoff in terms of sensitivity, as was the case only for Aβ42 over ttau, would decrease the number of subjects needed to scan to reach a prespecified number of positive cases.

### Added value of Aβ isoforms Aβ38 and Aβ40

The shorter isoforms Aβ38 and Aβ40 on their own had no diagnostic value to discriminate preclinical AD, in line with previous studies in cognitively intact healthy controls [14], and also in clinical AD patients [26]. In the context of preclinical AD, the added value of the Aβ isoforms mainly occurred when used for calculating ratios. The ratio over Aβ40 performed better than Aβ42 alone if a high specificity was required (Table 3).

The impact of using Aβ isoforms on the clinical accuracy is linked in part to the context of use. In some studies comparing clinical AD with healthy controls, the ratio of Aβ42 over Aβ38 or Aβ40 improved overall diagnostic accuracy [27, 28], but in others it did not [26, 29]. For the discrimination between clinically probable AD and non-AD dementias, the discriminative value of Aβ42/Aβ40 was similar to that of the ratio over ttau and better than Aβ42 alone [30, 31]. In the MCI stage of the disease, the predictive value for progression to dementia over a 4-year interval was higher for Aβ42/Aβ40 (AUC = 0.866) than for Aβ42 alone (AUC = 0.768) [13]. In our study, Aβ42/Aβ40 still allowed acceptable sensitivity for a specificity of 90 %, and more so than Aβ42 in isolation.

The reason why ratios perform better than Aβ42 in isolation may be methodological: the normalization procedure may remove a portion of the preanalytical and analytical variability in the measurement of the protein levels that is in itself unrelated to AD. In that case, as better standards become available for Aβ42 measurement, the benefit of using ratios will diminish. Alternatively, the ratio may perform better than Aβ42 for biological reasons. Many autosomal dominant forms of AD are associated with an increase in the ratio of Aβ42 over Aβ40 [32, 33]. Others, such as the Dutch and the Arctic APP mutation, are associated with the inverse effect [32]. If the driving force in the initial phases of sporadic AD is related to disequilibrium between different isoforms rather than the absolute amount of Aβ42 on its own, this could theoretically explain why the ratio would be better.

### Ratio of Aβ42 over ttau

For a fixed specificity of 95 %, the highest sensitivity (71 %) was obtained for Aβ42 over ttau. Generally, ttau is thought to reflect neuronal loss. Adding the separate measurement of a biomarker that increases with the intensity of the neurodegenerative process may enhance specificity because AD is a multidimensional disease [34, 35] so that adding a second dimension (neuronal loss) improves accuracy of classification. The added value of combining Aβ42 with ttau for the definition of preclinical AD is in line with the International Working Group IWG-2 criteria for preclinical AD which advocate for the combined use of both Aβ42 and ttau or ptau [3].

### CSF cutoff value for positive classification

The optimal Aβ42 cutoff value for the INNOTEST assay was higher than what is commonly applied in clinical practice. Previous studies have also suggested that cutoff values derived from studies in patients with more or less advanced stages of AD versus controls may not be entirely appropriate for distinguishing amyloid-positive from amyloid-negative healthy cognitively intact older adults [14, 36]. This has implications for clinical trials aiming to sensitively select cognitively intact subjects with increased Aβ aggregation [36].

### Potential study limitations

Our study has some limitations. The sample size was relatively low and the number of amyloid-positive cases was relatively small. Larger studies of preclinical AD will be needed to confirm the estimates of sensitivity and specificity. The low sample size is related to the strict inclusion and exclusion criteria. All subjects were recruited from the community and volunteered for the lumbar puncture purely for research purposes and were informed beforehand that they would not receive any feedback about their proper CSF results. We also applied strict criteria regarding the normality of the neuropsychological test scores. Given the small sample size we were careful to base our conclusions on the most robust findings: we applied strict correction for multiple comparisons and ascertained that our findings were replicable across different assay types and did not depend on small variations of the PET cutoff value within the range of the known test–retest variability of ^18^F-flutemetamol PET. For all these reasons we consider our results reliable despite the relatively small sample size, in particular the comparisons between AUC analyses. The repercussions of fixing specificity at 90–95 % on sensitivity have to be interpreted more cautiously: given the relatively low number of true positives, a change in classification of an individual case from positive to negative may lead to a disproportionately large decrease in sensitivity.

A community-recruited cohort is not equivalent to a population-based cohort and could be prone to a selection bias, targeting subjects concerned about their cognition, subjects who were more educated or more mobile, etc. We were careful not to mention memory, cognition, or related terms in our advertisement. The research question at hand, namely the comparison between CSF and PET for the research definition of preclinical AD, is most pertinent for a community-recruited setting: clinical trials targeting preclinical AD will generally not be based on population-based nor on memory clinic-based cohorts but on community-recruited cohorts. There was no evidence for a positive selection bias compared with other community-recruited cohorts. If anything, also taking into account the prior stratification for APOE ε4 in our study, our percentage of amyloid-positive cases was lower than in most other community-recruited studies [37]. In a population-based cross-sectional study of cognitively intact adults 50–89 years old, the frequency of amyloid-positive individuals was similar to that in our study [38]. The proportion of subjects who confirmed subjective memory complaints was also not particularly elevated compared with community-based [39, 40] or population-based studies [41].

Our standard of truth was ^18^F-flutemetamol positivity based on an autopsy-validated cutoff value. We have previously demonstrated a high concordance between ^18^F-flutemetamol and ^11^C-Pittsburgh Compound B for the definition of preclinical AD [42]. The autopsy study covered the different Thal stages 1–5 [43]. However, it remains possible, theoretically, that if measured in a population restricted to cognitively intact older adults, the cutoff value for distinguishing moderate to high neuritic amyloid density from sparse to low density may be lower than what is found in a mixed group including patients with advanced dementia along with dementia-free individuals [43]. According to the current study logic, a case who has low Aβ42 values but a normal ^18^F-flutemetamol value would be considered a false-positive. We cannot, however, exclude that this case is in a preclinical state preceding amyloid deposition detectable by PET [14]. In the selection of subjects who have increased risk of amyloid deposition but who have not yet reached the amyloid positivity threshold, there could still be a role for Aβ isoforms beyond Aβ42, although this remains to be demonstrated. The specificity required to define preclinical AD based on biomarkers will depend on the type of clinical trial. Different therapeutic strategies may target different preclinical stages of the disease. Our findings are mainly relevant for those trials that target a phase where amyloid aggregation has already occurred and where a marker must be selected, CSF versus amyloid PET.

## Conclusion

For selection of subjects with increased PET amyloid load, if a high specificity is required, our data support the use of Aβ42 over ttau rather than using Aβ42 alone or the ratios to other Aβ isoforms.

## Abbreviations

Aβ: Amyloid beta
AD: Alzheimer’s disease
AIC: Akaike information criterion
APOE: Apolipoprotein E
AUC: Area under the receiver operating characteristic curve
BDNF: Brain-derived neurotrophic factor
CDR: Clinical Dementia Rating
comp: Composite cortical volume of interest
CSF: Cerebrospinal fluid
ELISA: Enzyme-linked immunosorbent assays
MCI: Mild cognitive impairment
MNI: Montreal Neurological Institute
MMSE: Mini Mental State Examination
MRI: Magnetic resonance imaging
NIA–AA: National Institute on Ageing–Alzheimer’s Association
PET: Positron emission tomography
ptau: ^181^Phospho-tau
ROC: Receiver operating characteristic curve
SD: Standard deviation
SUVR: Standardized uptake value ratios
ttau: Total tau
